# Interaction between dissolved organic carbon and fungal network governs carbon mineralization in paddy soil under co-incorporation of green manure and biochar

**DOI:** 10.3389/fmicb.2023.1233465

**Published:** 2023-08-22

**Authors:** Kun Cheng, Xiaoyue Wang, Libo Fu, Wei Wang, Ming Liu, Bo Sun

**Affiliations:** ^1^Key Laboratory of Poyang Lake Basin Agricultural Resource and Ecology of Jiangxi Province, College of Land Resource and Environment, Jiangxi Agricultural University, Nanchang, China; ^2^State Key Laboratory of Soil and Sustainable Agriculture, Institute of Soil Science, Chinese Academy of Sciences, Nanjing, China; ^3^Agricultural Environment and Resources Institute, Yunnan Academy of Agricultural Sciences, Kunming, China

**Keywords:** C mineralization, dissolved organic carbon, green manure, biochar, fluorescence spectra, microbial communities, co-occurrence network

## Abstract

Legume crops in rice cultivation are typically rotated and incorporated into the soil as green manure to improve soil fertility. Biochar has recently been co-incorporated with green manure to simultaneously stimulate soil organic carbon (SOC) mineralization and increase carbon (C) sequestration. However, few studies examine the effects of the co-incorporation of biochar and green manure on C cycling and the underlying microbial mechanisms in paddy fields. In this study, the effects of the co-incorporation of green manure and biochar on C mineralization, dissolved organic carbon (DOC) characteristics, and microbial community structures were investigated. A pot study was conducted with three treatments: inorganic NPK (NPK), inorganic NPK + green manure (GM), and inorganic NPK + green manure + biochar (GMC). Organic amendments significantly increased cumulative C mineralization, with amounts in the order GMC (3,434 mg·kg^−1^) > GM (2,934 mg·kg^−1^) > NPK (2,592 mg·kg^−1^). Fertilizer treatments had similar effects on DOC concentrations, with amounts in the order GMC (279 mg·kg^−1^) > GM (255 mg·kg^−1^) > NPK (193 mg·kg^−1^). According to fluorescence spectra, the highest microbial humic acid-like fraction and biological index were also in GMC. Co-incorporation of green manure and biochar shifted the composition of bacterial and fungal communities but more importantly, increased fungal network complexity and decreased bacterial network complexity. The increase in fungal network complexity with the increase in DOC concentrations and microbially derived components was the dominant factor in promoting C mineralization. Overall, this study reveals the underlying biochemical mechanism, the interaction between DOC and fungal network of C cycling in paddy soil under the co-incorporation of green manure and biochar management, and provides fundamental knowledge for exploring effective approaches to improve soil fertility and health in the future.

## 1. Introduction

Soil organic carbon (SOC) and its mineralization rate are important indicators reflecting soil fertility and health, with high carbon (C) content and mineralization rates typically considered desirable properties (Lehmann et al., 2020). Paddy soil in China is at risk of losing SOC due to soil acidification and compaction resulting from the excessive use of inorganic fertilizers (Chen et al., 2016; Xia et al., 2020). In crop rotation systems, green manure incorporation is a common practice to increase C sequestration and maintain soil fertility (Yuan and Yue, 2012; Gao et al., 2018). However, over 70% of green manure can be decomposed within a month due to its high content of labile C (Zhu et al., 2014). Consequently, it may take more than a decade to increase SOC by 1 g·kg^−1^ solely through green manure incorporation (Pan et al., 2010; Kamran et al., 2021; Zhang et al., 2023). Compared with green manure, biochar is very stable in soil and has been recognized as one of the best approaches to sequestering C in soils (Han et al., 2020; Liu et al., 2022). The co-incorporation of green manure and biochar can effectively simultaneously stimulate C turnover and enhance C sequestration in uplands (Liu Y. L. et al., 2021). However, few studies focus on the effects of the combined incorporation of green manure and biochar on C cycling and the underlying microbial mechanisms in paddy fields.

Organic amendments affect C cycling by regulating SOC quantity and quality and affecting microbial community structures (Thieme et al., 2019; Wu et al., 2023). Dissolved organic carbon (DOC) is the most active SOC fraction and has a vital role in C cycling. Changes in DOC concentrations and composition can directly affect C mineralization (Chow et al., 2006). Green manure application is known to stimulate C mineralization by increasing DOC concentration and introducing labile C sources (Gao et al., 2018). Biochar contains large recalcitrant organic compounds, such as aromatic compounds (Huang et al., 2019; Cui et al., 2020). When green manure is co-incorporated with biochar, contents of both labile and recalcitrant C are expected to increase and DOC composition is expected to change. However, how the co-incorporation of green manure and biochar affects DOC autochthonous biological index and humus index remains unknown. Furthermore, few studies reported how changes in DOC concentration and composition induced by the co-incorporation of green manure and biochar regulate C mineralization.

Carbon mineralization is a microbially mediated process (Huang et al., 2021), and exogenous organic materials of different quality lead to differences in microbial succession and thus in community structure. Green manure application promotes the abundance of *Alphaproteobacteria* and other copiotrophs (Yang et al., 2023), whereas biochar application increases the fungi-to-bacteria ratio as well as the abundance of *Actinobacteria* and other oligotrophs (Wang N. Y. et al., 2022). More importantly, as a “broad process,” C mineralization involves many functionally and taxonomically diverse microorganisms (Schimel and Schaeffer, 2012; Whitman et al., 2016; Wagg et al., 2019). Previous publications suggest that C mineralization is influenced by the interaction of microorganisms (Banerjee et al., 2016; Wang et al., 2021). The co-occurrence network has been increasingly used to explore potential interactions between species (Barner et al., 2018; Guseva et al., 2022). Network topological properties can reveal patterns of interaction and functional diversity of microbial communities (Gamfeldt and Roger, 2017). Organic amendments have been reported to affect network complexity (Ling et al., 2016), and increased network complexity may contribute to improved C utilization efficiency (Xu et al., 2023).

Based on the background of the above discussion, the aim of this study was to explore the effects of the co-incorporation of green manure (*Astragalus sinicus* L.) and biochar on C mineralization and the underlying mechanism. A one-year pot experiment was conducted, and soil samples collected at the rice (*Oryza sativa* L.) filling stage were analyzed for cumulative C mineralization (*Cum*), DOC concentrations and fluorescence components, and composition and network structures of bacterial and fungal communities. The hypotheses of the study were the following: (1) compared with no organic amendments and only application of green manure, the co-incorporation of green manure and biochar would increase DOC concentrations and the proportion of microbially derived C; (2) co-incorporation of green manure and biochar would lead to higher bacterial and fungal network complexity than that in other treatments; and (3) DOC characteristics and microbial network complexity would be dominant factors regulating C mineralization.

## 2. Materials and methods

### 2.1. Experimental design and samples collection

A pot experiment simulating crop rotation with Chinese milk vetch (CMV, *Astragalus sinicus* L. “Xiangzi No.1”) and rice (*Oryza sativa* L. “Y liangyou 2108”) was conducted from October 2018 to October 2019 in the greenhouse of the Chongming experimental station of the Yunnan Academy of Agricultural Sciences (25° 21′ 22″ N, 103° 01′ 48″ E). The soil was collected from the Ap horizon (0–20 cm) in a nearby rice field. The paddy soil was classified as an Anthrosols according to the FAO soil taxonomy. The region has a tropical monsoon climate with an average annual temperature of 15°C and precipitation of 1,035 mm. After the removal of visible plant tissue, the soil was air-dried, sieved through a 2 mm mesh, and fully mixed. Mixed soil was placed in plastic pots, and each pot contained 3.0 kg of soil. Soil properties are shown in Supplementary Table S1.

The experiment had three fertilization treatments: (1) NPK: chemical fertilizer N (N at 0.10 g·kg^−1^ dry soil), (2) GM: chemical fertilizer N and green manure (N at 0.08 g·kg^−1^ dry soil + CMV fresh green manure at 4.06 g·kg^−1^ dry soil), and (3) GMC: chemical fertilizer N, green manure and biochar (N at 0.08 g·kg^−1^ dry soil + CMV fresh green manure at 4.06 g·kg^−1^ dry soil + biochar at 20 g·kg^−1^ dry soil). In addition, P_2_O_5_ (0.05 g·kg^−1^) and K_2_O (0.06 g·kg^−1^) were added in all treatments. The fertilizers used were urea (N 46%), calcium superphosphate (P_2_O_5_ 12%), and potassium chloride (K_2_O 60%). The properties of green manure and biochar are provided in Supplementary Table S2. Each treatment had eight replicates, with each replicate represented by one plastic pot. Chinese milk vetch was sown in October 2018, and in April 2019, and CMV at the blooming stage, approximately 1 month before the middle rice transplantation, was incorporated into the soil as green manure in GM and GMC treatments. Biochar in GMC and chemical fertilizer in all three treatments were incorporated into the soil at the same time as the CMV, with the water layer kept at 2–3 cm. The soil was drained for a week at the tillering stage and for 2 weeks before harvest. In August 2019, at approximately 90 d after middle rice transplantation, five soil cores were randomly sampled from each pot and then fully mixed to serve as one composite sample. Fresh samples were placed on ice and immediately transported to the laboratory. Soil samples were divided into three parts for soil physicochemical analysis, organic C mineralization, and DNA extraction, respectively.

### 2.2. Cumulative carbon mineralization

Soil organic C mineralization was measured in microcosm incubation experiments using an alkali absorption method according to Jiang et al. (2018). Briefly, 30.0 g of each air-dried soil sample was adjusted to 65% moisture content of field capacity and then after 3 d of pre-incubation, incubated in 500 mL jars for 30 d at 25°C. Soil samples were replicated six times, and six control jars (pure silica sand without soil) were used to measure background CO_2_ concentration. In each jar, a glass vial containing 0.5 mol·L^−1^ NaOH was placed to trap CO_2_ emitted from the soil. After incubating for 1, 3, 5, 7, 10, 15, 20, 25, and 30 d, the amount of CO_2_ trapped in the alkali solution was measured by titration. The remaining alkali was titrated to pH 7 with 0.4 mol·L^−1^ HCl after precipitating carbonate with 20 mL of 1 mol·L^−1^ BaCl_2_ solution. After each sampling, incubation jars remained open for 1 h for gas exchange and to reach ambient O_2_ level, and glass vials were refilled with new NaOH solutions. Incubation jars containing wetted soil samples were weighed before incubation, and distilled water was added when needed to maintain soil moisture. Finally, mineralization rates were calculated on the basis of daily CO_2_ emissions. Cumulative C mineralization (*Cum*) was the sum of the mineralization in each stage of the incubation (mg CO_2_-C per kg soil).

### 2.3. Dissolved organic carbon extraction and characterization

Soil DOC was extracted with Milli-Q water (1:10 w/v) by mixing on a reciprocating shaker (ZWYR-4912, Zhicheng, China) for 2 h at 180 rpm at 25°C (Li et al., 2020). Supernatants were collected as DOC solutions after centrifugation at 8,000 rpm for 10 min and filtration through a 0.45 μm (Fisher) microporous filter membrane. Dissolved organic C concentration was measured on a total organic carbon analyzer (multi N/C3100, Analytik Jena, Germany). Excitation-emission fluorescence spectra (EEMs) were determined using a photometer (F7000 FL, Hitachi, Japan). To obtain EEMs, excitation (Ex) wavelengths were set to increase gradually from 200 nm to 450 nm in increments of 5 nm, and the emission (Em) was measured at each excitation wavelength from 250 nm to 600 nm with 1 nm increments. Blank EEMs were obtained using Milli-Q water in the same way. To minimize instrument-specific effects on EEMs and eliminate scatters, first, the EEMs of samples and blank were normalized to the Raman peak area (Ex = 350 nm) to eliminate the fluorescence intensity differences caused by the fluorescence meter. Rahman normalized blank EEMs were then subtracted from sample EEMs to remove Rayleigh scatters and obtain corrected EEMs.

Parallel factor analysis (PARAFAC) was used to resolve the EEMs into different components using the package “StaRdom” in R (Pucher et al., 2019; Liu J. Z. et al., 2021). Non-negative constraints were applied to the parameters to allow only realistic results on the chemical composition. A three-component model was validated with the split-half analysis program of Murphy et al. (2013). The model explained 99.5% of the variance in the dataset. The Fmax values were the fluorescence of each component in each sample at their respective excitation and emission maximums and were used to quantify the component. For quantitative comparisons with published data, the components identified as constituents were searched using the OpenFluor online database of automated fluorescence spectra of organic compounds in the environment (http://www.OpenFluor.org). OpenFluor defines a similar spectrum to that having a Tucker congruence (TCC) greater than 0.95 on both excitation and emission spectra, and possible sources of the identified components were obtained based on the OpenFluor search results.

Biological index (BIX) is used to evaluate the proportion of microbially derived carbon in DOC (Wang et al., 2015), and the humification index (HIX) represents the degree of DOM humification (Wang et al., 2013). These indexes are calculated by the following equations:


(1) BIX=Fem380/ex310/Fem430/ex310

(2) $$\begin{array}{l} \text{HIX}=F\text{em}\;\left(435-480\right)/\text{ex}254/\\ \;\left(F\text{em}\;\left(300-345\right)/\text{ex}254+F\text{em}\;\left(435-480\right)/\text{ex}254\right) \end{array}$$

where the *F*_em/ex_ represents the fluorescence intensity at a specific emission wavelength (em) or an emission wavelength (em) range which excited at a specific excitation wavelength (ex).

### 2.4. DNA extraction and MiSeq sequencing

Soil DNA was extracted from 0.5 g samples using a FastDNA Spin Kit (MP Biomedicals, Santa Ana, CA, United States) following the manufacturer’s instructions. After PCR amplifications by the bacterial universal primers 341F (5′-CCTAYGGGRBGCASCAG-3′) and 806R (5′-GGACTACHVGGGTWTCTAAT-3′) (Gardes and Bruns, 1993) and the fungal universal primers ITS1F (5′-CTTGGTCATTT AGAGGAAGTAA-3′) and ITS2R (5′-GCTGCGTTCTTCA TCGATGC-3′) (Lee et al., 2012). Samples were sent to Shanghai Biozeron Co., Ltd. (Shanghai, China) for sequencing via MiSeq (Illumina). After sequencing, raw data were trimmed with the program Trimmomatic, including quality trimming and chimera removal, to obtain paired-end clean reads. Then, clean paired-end reads were aligned by FLASH (V1.2.11) and filtered to remove barcodes and primers with Mothur (V1.42.1). Finally, operational taxonomic units (OTUs) were clustered by the uparse method via Usearch at 97% identity. The SILVA database (v138.1) was used to align bacterial 16S data (Pruesse et al., 2007), and the UNITE database (v8.2) was used to align fungal ITS data (Koljalg et al., 2013). In all samples, 9,087 and 2,264 OTUs were identified from16S rRNA and ITS data, respectively. Sequencing data were deposited in the Sequence Read Archive (SRA) in NCBI under Bioproject PRJNA933112.

### 2.5. Data analysis

Analysis of variance (ANOVA) and Tukey tests were used to determine differences in soil properties and C mineralization among different treatments using R (v 4.1.3, R Development Core Team). All data are presented as the mean ± standard error. Principal Coordinates Analysis (PCoA) and Adonis test were used to explore the changes in bacterial and fungal community structures among different treatments via the cmdscale function in the vegan package.

Package “Hmisc” was used to construct co-occurrence networks based on a Spearman correlation matrix. The OTUs were filtered by their presence in more than half of the samples, and 2,895 and 333 OTUs were included in bacterial and fungal network analyses, respectively. Then all Spearman correlations between OTUs were calculated, and *p*-values were adjusted by the Benjamini and Hochberg false discovery rate (FDR) test, with adjusted *p*-values having a 0.01 cutoff. The focus was only on microbial taxa that were highly correlated with one another because that indicated that they had strong coexistence within a community (Herren and Mcmahon, 2018). Then, Gephi 0.9.3 was used to visualize the final networks, calculate network topological characteristics and construct modules. Mantel test in the package “vegan” in R (v4.1.3) was used to calculate the Pearson correlation coefficients between module eigengenes and soil properties and cumulative C mineralization (*Cum*).

Random forest modeling was used to assess the importance of predictors of C mineralization using the package randomForest in R (v4.1.3). The significance of the model and predictor importance were determined using the packages “A3” and “rfPermute,” respectively, modified according to Jiao et al. (2018). Potential impact factors were then classified into two categories: DOC characteristics and microbial community structures. Canonical correlation analysis (CCA)-based variance partitioning analysis (VPA) was used to determine the relative importance of the two categories on C mineralization using the “vegan” package in R (v4.1.3).

## 3. Results

### 3.1. Soil properties, carbon mineralization, and rice biomass

**Figure 1:**
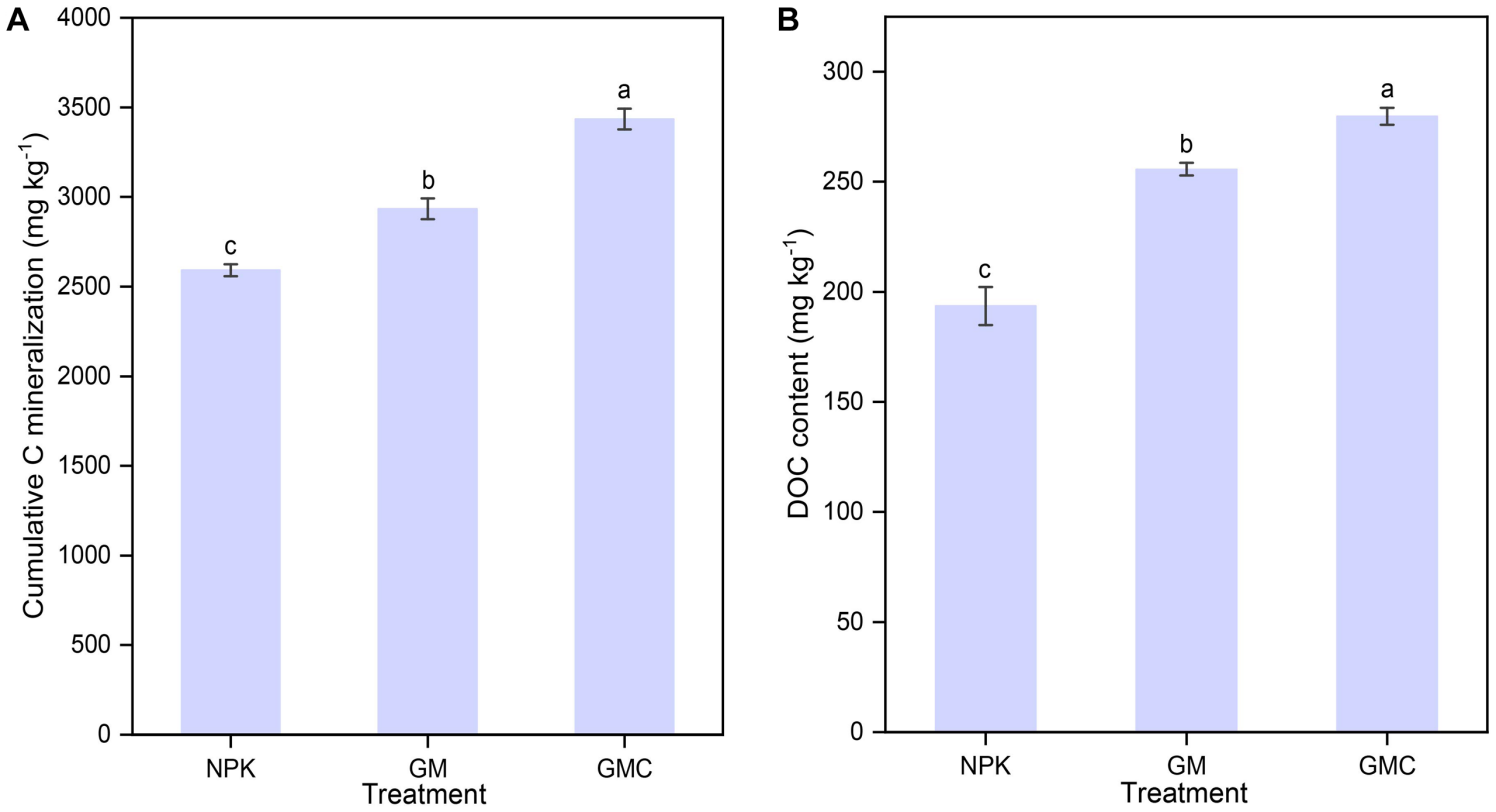
Effects of fertilizer amendments on the cumulative carbon mineralization **(A)** and DOC concentration **(B)**. Values are the mean ± standard errors. Different lowercase letters indicate significant difference under *p* < 0.05. NPK, inorganic NPK fertilizer; GM, NPK + green manure; GMC, NPK + green manure + biochar.

Organic amendments significantly affected the soil nutrient contents (Table 1). The highest SOC, total N (TN), and total P (TP) contents were in GMC. Whereas the contents of most soil nutrients were not significantly different between GM and NPK. In addition, organic amendments also significantly increased *Cum*, with GMC (3,434 mg kg^−1^) > GM (2,934 mg kg^−1^) > NPK (2,592 mg kg^−1^) (*p* < 0.05; Figure 1A). Similarly, GMC had the highest DOC concentrations, followed by that in GM and NPK (*p* < 0.05; Figure 1B). Moreover, the aboveground biomass fractions of stem, leaf, and grain, were the highest in GMC and the lowest in NPK, whereas there were no significant differences in root biomass among treatments (Supplementary Table S3).

**Table 1:** Soil properties at rice filling stage.

Treatment	pH	SOC (g·kg^−1^)	NH_4_^+^-N (mg·kg^−1^)	NO_3_^−^-N (mg·kg^−1^)	TN (g·kg^−1^)	C/N	TP (g·kg^−1^)	AP (mg·kg^−1^)
NPK	7.72 ± 0.07a	21.04 ± 0.42ab	13.66 ± 0.74a	0.68 ± 0.07a	2.11 ± 0.03b	9.93 ± 0.23a	0.69 ± 0.08b	101.65 ± 4.55a
GM	7.78 ± 0.12a	20.74 ± 0.69b	11.29 ± 0.44b	0.50 ± 0.03a	2.10 ± 0.07b	9.88 ± 0.59a	0.77 ± 0.25ab	98.32 ± 4.37a
GMC	7.77 ± 0.05a	22.43 ± 1.42a	12.20 ± 0.48ab	0.51 ± 0.03a	2.24 ± 0.05a	9.98 ± 0.49a	1.00 ± 0.15a	101.47 ± 4.07a

Values within the same column followed by different letters indicate significant differences at *p* < 0.05. NPK, inorganic NPK fertilizer; GM, NPK + green manure; GMC, NPK + green manure + biochar; SOC, soil organic carbon; NH4^+^-N, ammonia nitrogen; NO_3_^−^-N, nitrate nitrogen; TN, soil total nitrogen; C/N, soil organic carbon to total nitrogen ratio; TP, soil total phosphorus and AP, soil available phosphorus.

### 3.2. Excitation-emission fluorescence spectra of dissolved organic carbon fractions

**Figure 2:**
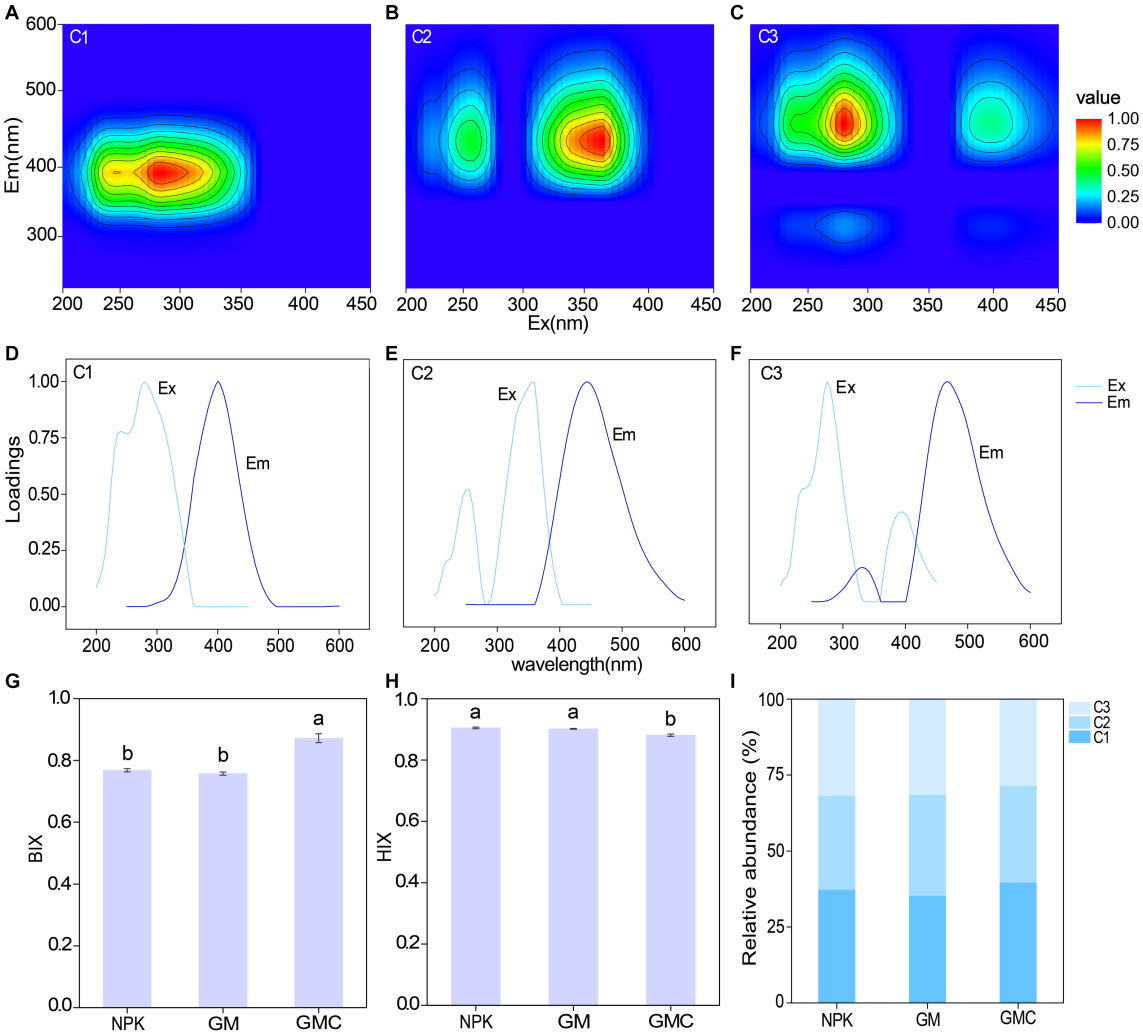
Parallel factor analysis of components extracted from fluorescence excitation (ex) – emission (em) matrix spectra of dissolved organic carbon in different fertilizer treatments. Top panels: contour plots of the components C1 **(A)**, C2 **(B)**, and C3 **(C)**; middle panels: Corresponding excitation (Ex) and emission (Em) spectral loadings of the model, **(D–F)**; bottom panels: effects of different fertilizer treatments on autochthonous biological index (BIX) **(G)**, humus index (HIX) **(H)** and relative abundances of different fluorescent components **(I)**. Different letters indicate significant differences at *p* < 0.05.

The fluorescence EEMs spectra of the solutions extracted from the three treatments were resolved into three components by the PARAFAC analysis (Figures 2A–F). According to fluorescence spectra and published data, the three major components were microbial humic acid-like substances (C1), and terrestrial humic acid-like substances (C2 and C3). Excitation (Ex) and emission (Em) peaks and characteristics of each component are summarized in Table 2. In addition, the relative abundance of C1 was significantly higher in GMC than in the other treatments, whereas C3 showed the opposite trend (Figure 2I). The biological index (BIX) was significantly higher in GMC than in the other treatments, whereas there were no significant differences in the humus index (HIX) among the treatments (Figures 2G,H).

**Table 2:** Characteristics of dissolved organic carbon components identified by excitation (ex)-emission (em) fluorescence spectral analysis.

Components	Peak location λex/em (nm)	Probable origin	Number of OpenFluorb matches	References
C1	280/401	Microbial humic acid-like	35	Lin and Guo (2020)
C2	355/443	Terrestrial humic acid-like	11	Murphy et al. (2011)
C3	275/468	Terrestrial humic acid-like	3	Amaral et al. (2021)

### 3.3. Microbial community diversity and composition

**Figure 3:**
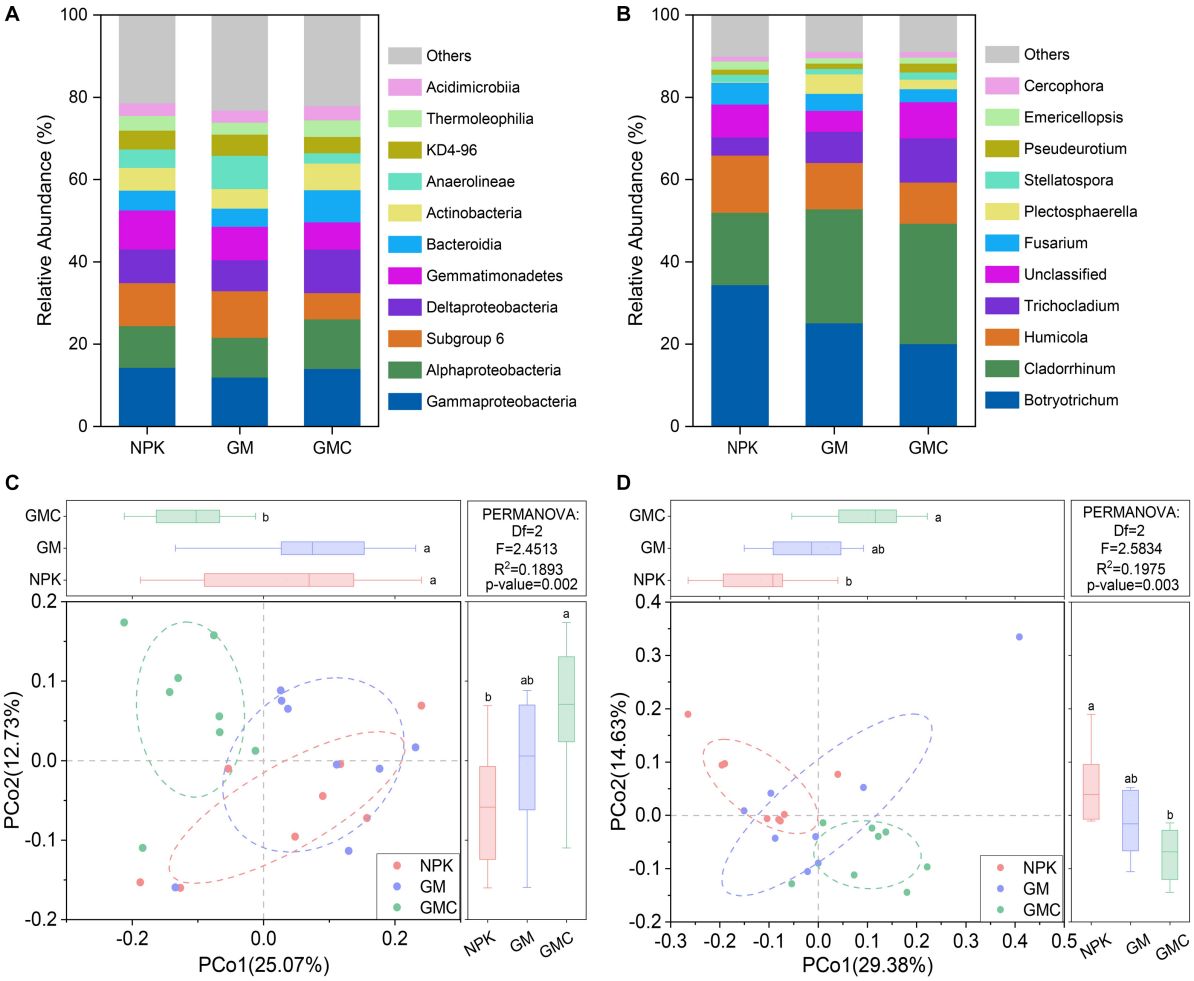
Relative abundances of major classes within the bacterial communities **(A)** and major genus within the fungal communities **(B)** under different fertilizer treatments. Principal coordinate analysis based on Bray-Curtis distance shows the effect of different fertilizer treatment on bacterial **(C)** and fungal **(D)** communities. NPK, NPK fertilization; GM, NPK fertilization + green manure; GMC, NPK fertilization + green manure + biochar. Different letters indicate significant differences at *p* < 0.05.

Bacterial communities were dominated by *Gammaproteobacteria* (13.4% on average), *Alphaproteobacteria* (10.6% on average), and Subgroup 6 of *Acidobacteria* (9.3% on average) (Figure 3A). Principal co-ordinates analysis (PCoA) indicated the composition of bacterial communities was altered by co-incorporation of green manure and biochar (*R*^2^ = 0.189; *p* < 0.01; Figure 3C). When considering the relative bacterial abundances, the relative abundances of *Gammaproteobacteria*, *Alphaproteobacteria*, *Deltaproteobacteria*, and *Bacteroidia* increased by co-incorporation of green manure and biochar, whereas those of Subgroup 6 of *Acidobacteria*, *Gemmatimonadetes*, and *Anaerolineae* decreased (*p* < 0.05; Figure 3A). In addition, the fungal communities were dominated by *Botryotrichum* (26.5% on average) and *Cladorrhinum* (24.8% on average) (Figure 3B). Similar to the bacterial community, the PCoA of fungal community compositions revealed significant (*R*^2^ = 0.197, *p* < 0.01) separation among NPK, GM, and GMC treatments (Figure 3D). The relative abundance of *Cladorrhinum* increased with organic amendments (GM and GMC), whereas that of *Botryotrichum* decreased with organic amendments, with abundance in the order NPK > GM > GMC (*p* < 0.05; Figure 3B).

### 3.4. Bacterial and fungal networks

**Figure 4:**
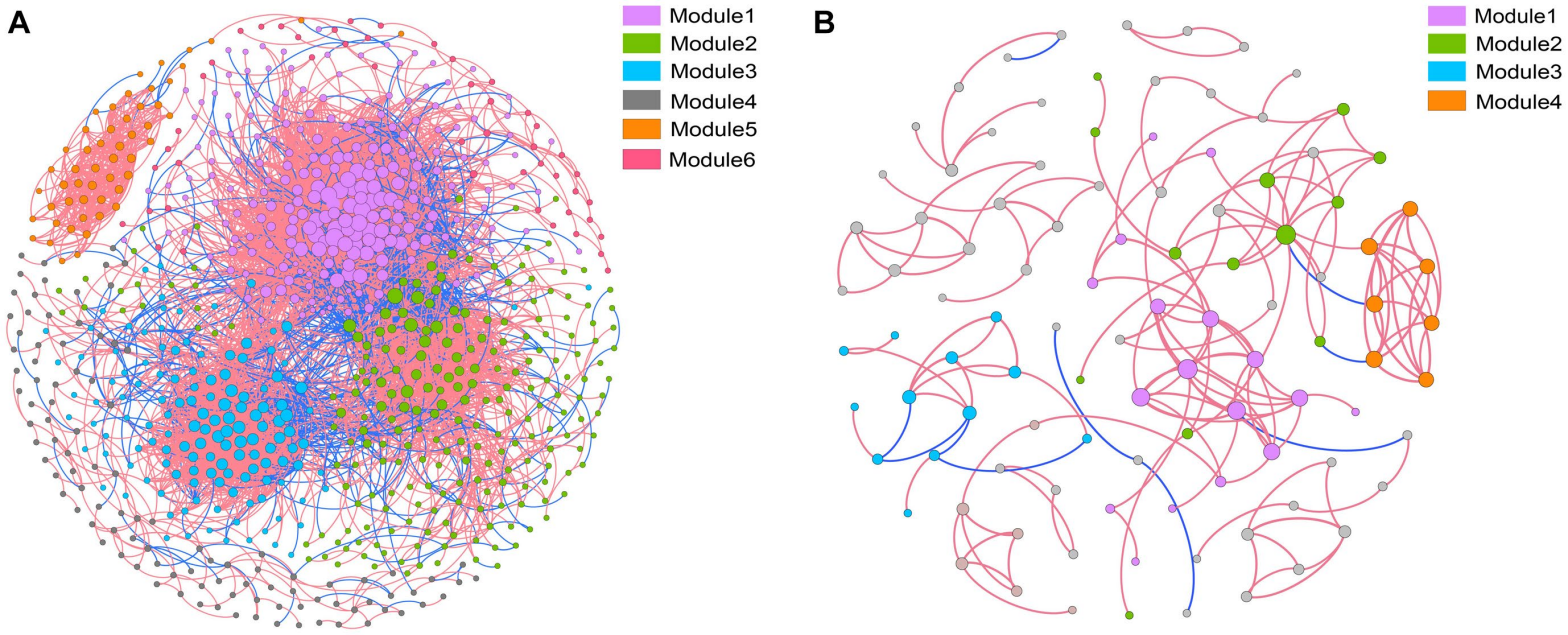
Co-occurrence networks and modular patterns of bacterial **(A)** and fungal **(B)** communities in response to different fertilizer treatments. A connection indicates a significant (*p* < 0.01) correlation between two operational taxonomic units. Modules in the networks are clusters of closely interconnected nodes. The size of each node reflects the number of connections (degree), and the thickness of each connection between two nodes (edge) reflects the value of the Spearman correlation coefficients. A red edge indicates positive interaction, and a blue edge indicates negative interaction.

**Figure 5:**
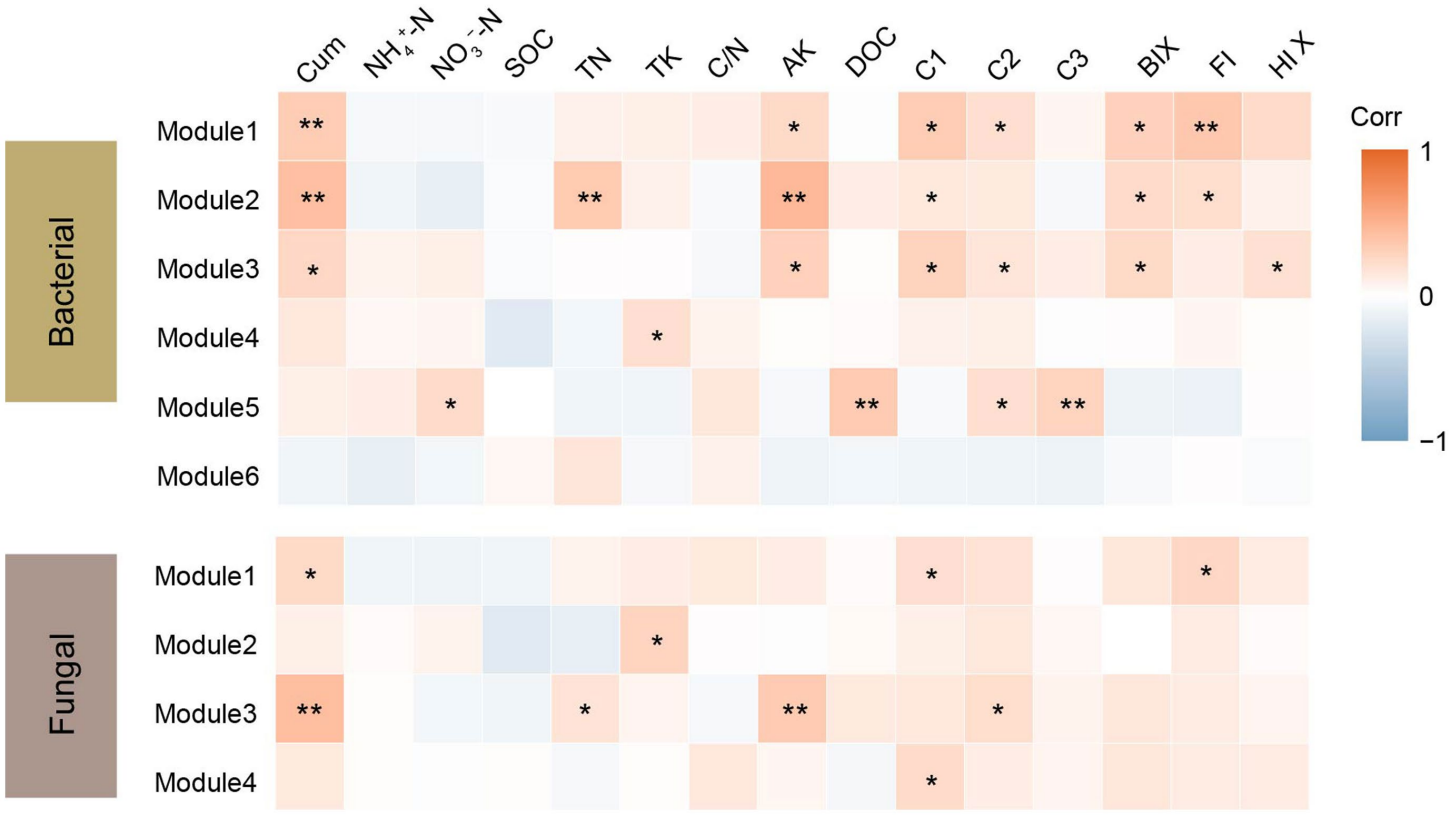
Correlation coefficients between bacterial and fungal module eigengenes, soil nutrients, dissolved organic carbon (DOC) content and components, fluorescence index and cumulative carbon mineralization (CO2) under different fertilizer treatments. Cum, cumulative carbon mineralization; NH_4_^+^-N, ammonia nitrogen; NO_3_^–^-N, nitrate nitrogen; SOC, soil organic carbon; TN, soil total nitrogen; TK, soil total potassium, C/N, soil carbon to nitrogen ratio; AK, soil available potassium; C1, C2 and C3, DOC components; BIX, biological index; FI, fluorescence index and HIX, humus index. *p < 0.05; **p < 0.01.

Co-occurrence networks were constructed to identify differences in co-occurrence patterns of soil bacterial (Figure 4A) and fungal (Figure 4B) communities in different treatments. For a meaningful comparison of network topological properties in individual treatments, subnetworks were generated from bacterial and fungal communities. Organic amendments significantly changed the topological properties of bacterial and fungal networks (Table 3). In bacterial networks, green manure application significantly increased the proportion of positive linear relationships and the network complexity, expressed as the average clustering coefficient (ACC) (Table 3). In fungal networks, positive interactions between species dominated the whole network. In addition, organic amendments significantly increased the network complexity (expressed as ACC), with complexity in the order GMC > GM > NPK. Bacterial and fungal networks were clustered into modules to identify significant module-environmental parameter relations. According to Mantel tests, most modules in bacterial networks were positively correlated with environmental parameters. Modules 1, 2, and 3 were positively correlated with *Cum* (cumulative C mineralization) and C1 of DOC (Figure 5). In fungal network modules, except for modules 1 and 3, there were few correlations between other modules and environmental parameters.

**Table 3:** Topological properties of bacterial and fungal subnetworks in different fertilizer treatments.

		NPK	GM	GMC
Bacterial	Nodes	646b	689a	676ab
Subnetwork	Edges	6021b	6788a	6200b
	Average clustering coefficients	0.39a	0.40a	0.38a
	Average degree	20.8a	21.2a	20.1a
	Positive correlation edges	5108b	5787a	5202b
	Negative correlation edges	913a	1001a	998a
Fungal	Nodes	88a	95a	90a
Subnetwork	Edges	138a	148a	141a
	Average clustering coefficients	0.43c	0.48b	0.56a
	Average degree	3.23b	3.28ab	3.31a
	Positive correlation edges	131a	140a	135a
	Negative correlation edges	7a	8a	6a

Values within the same column followed by different letters indicate significant differences at *p* < 0.05. NPK, inorganic NPK fertilizer; GM, NPK + green manure; GMC, NPK + green manure + biochar.

### 3.5. Potential dominant factors affecting carbon mineralization

**Figure 6:**
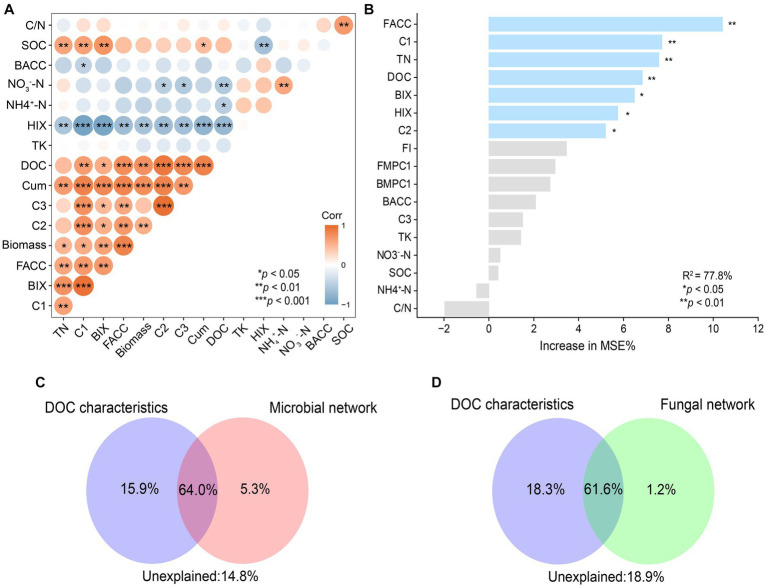
The correlation between biotic and abiotic factors **(A)**. Red indicates significant positive correlations. Blue indicates significant negative correlations. Significance levels of each predictor: *p < 0.05, **p < 0.01 and ***p < 0.001. Main predictors of soil organic carbon mineralization **(B)**. The figure shows the Random Forest mean predictor importance (% of increase of MSE) of soil variables drivers and microbial network (average clustering coefficients) on SOC mineralization. Proportion of variation in SOC mineralization explained by dissolved organic carbon, microbial **(C)** and fungal **(D)** using canonical correlation analysis based on variation partitioning analysis (VPA). Corr, correlation; Biomass, rice biomass; Cum, cumulative C mineralization; BACC, average clustering coefficient of bacterial subnetworks; FACC, average clustering coefficient of fungal subnetworks.

To determine the dominant factors affecting C mineralization, correlation, random forests, and CCA based-VPA were applied. Firstly, the correlation analysis showed that rice biomass was positively correlated with *Cum*, DOC concentrations, and relative abundance of C1 and that *Cum* was positively correlated with TN, DOC concentrations, the relative abundance of C1(Component 1), and ACC (average clustering coefficients) of fungal networks (Figure 6A). In addition, the ACC of a fungal network was positively correlated with the relative abundance of C1. Random forests were constructed to evaluate the importance of individual factors on *Cum* (Figure 6B). Among all the factors, ACC of fungal networks and relative abundance of C1 were the two most important predictors of C mineralization, followed by TN, DOC concentrations, and indexes of DOC fluorescence spectra (*p* < 0.01). Then, the important predictors were classified into two categories: DOC characteristics (represented by DOC C1 and DOC concentrations) and microbial networks (represented by ACC of bacterial and fungal networks). Further, VPA was used to evaluate the relative importance of DOC characteristics, microbial networks and their interaction (Figures 6C,D). The interaction effect of the two categories explained approximately over 60% of the variation in C mineralization. In addition to the interaction effect, DOC characteristics (15.9%) explained a larger proportion of the variation in C mineralization than that of the microbial network (5.3%) (Figures 6C,D). Overall, it was found that ACC of the fungal network alone explained 90.6% of the variation in C mineralization due to microbial community, and DOC concentrations and C1 of DOC accounted for 50.2% and 43.5%, respectively, of the variation in C mineralization due to DOC.

## 4. Discussion

### 4.1. Effects of co-incorporation of green manure and biochar on dissolved organic carbon content and compositions

As hypothesized, compared to other treatments, the co-incorporation of green manure and biochar had greater effects on DOC concentrations and the proportion of microbially derived C, represented by microbial humic acid-like substances (C1) and BIX (Figures 1B, 2I). The increased DOC in GMC was likely not directly from the soluble C fraction in biochar because the biochar DOC concentrations was very low (Supplementary Table S2), and soil samples were collected 90 d after the biochar application. Instead, it was highly likely that biochar application increased microbial activity and biomass by providing habitats and increasing soil porosity (Chen et al., 2019). An increase in microbial biomass and activity can stimulate the degradation of large biopolymers into small soluble molecules and therefore increase the DOC concentrations. In addition, an increase in microbial activity can also increase available nutrient contents and promote rice growth (Supplementary Table S3), consequently leading to an increase in the abundance of rhizodepositions (Eisenhauer et al., 2017). Rhizodeposits can be further adsorbed by biochar and reduced losses from leaching (Joseph et al., 2021). The correlation analysis confirmed that DOC concentrations was positively correlated with rice biomass (Figure 6A).

Compared with the other treatments, the co-incorporation of green manure and biochar also increased the relative abundance of the microbial humic acid-like component (C1). In addition, the BIX was increased from 0.76 to 0.87 with the co-incorporation of green manure and biochar (Figure 2G). The BIX represents the autochthonous biological activity of DOC. A high BIX value (>0.8) indicates autochthonous origins, i.e., microbially derived materials, whereas a low BIX value (< 0.8) indicates allochthonous origins, i.e., exogenous organic materials (Catalan et al., 2014). The increase in microbial humic acid-like components and BIX suggested the proportion of microbial-derived DOC increased (Wang et al., 2015; Zhang et al., 2017). The observed increases may be attributed to the fact that biochar enhances microbial biomass and activities by promoting oxygen availability, particularly in nutrient-rich soils (Zhang et al., 2018 Pokharel et al., 2020). Therefore, in GMC, it was highly likely that increases in exocellular enzyme activity by biochar and green manure promoted the degradation of plant residues, such as green manure and root exudates, and decrease the abundance of exogenous organic materials. Meanwhile with sufficient labile C from green manure, increases in microbial biomass and assimilative activity by biochar could lead to the accumulation of microbial necromass, i.e., microbially derived carbon, and result in a shift in DOC composition toward autochthonous origins.

### 4.2. Effects of co-incorporation of green manure and biochar on soil microbial communities

The co-incorporation of green manure and biochar significantly shifted the composition of microbial communities and changed network topological properties (Figures 3, 4). Firstly, co-incorporation of green manure and biochar primarily promoted the relative abundance of fast-growing copiotrophic gram-negative bacteria, such as *Alphaproteobacteria* and *Deltaproteobacteria*, because of the sufficient liable C sources from green manure application (Almagro et al., 2021). Meanwhile, the relative abundances of some slow-growing, oligotrophic gram-positive bacteria also increased in GMC, such as *Bacteroidia* and *Actinobacteria*, likely because of the prevalence of large organic compounds from biochar application (Cui et al., 2020). In addition, although both belonged to saprophytic fungi, *Cladorrhinum* increased, whereas that of *Botryotrichum* decreased under the co-incorporation of green manure and biochar treatments (Figure 3B). *Botryotrichum* can produce some secondary metabolites that are unfavorable for plant growth (Elkhateeb and Daba, 2022). Therefore, the decrease in abundance *Botryotrichum* suggested that the co-incorporation of green manure and biochar could potentially decrease crop morbidity and increase soil health.

Whereas the increase in fungal network complexity (expressed by the ACC) with co-incorporation of green manure and biochar provided partial support for the second hypothesis of the study, the lowest bacterial network complexity with co-incorporation of green manure and biochar did not support the hypothesis. The decrease in bacterial network complexity could be related to the co-incorporation of green manure and biochar increasing the abundance of both copiotrophs and oligotrophs, as discussed above. The corticotrophs likely used the labile C from green manure, and the oligotrophs likely mineralized soil recalcitrant C from biochar (Ling et al., 2022). Therefore, the cooperation between species decreased (Table 3). To explain the increase in fungal network complexity, one possible reason is that biochar can increase the fungi-to-bacteria ratio, as well as fungal activity (Wang X. C. et al., 2022). In addition, fungal hyphae can more easily inhabit the relatively large nutrient-rich pores of biochar than bacteria (Joseph et al., 2021). With increases in the density of fungi colonizing large pores of biochar, interactions between fungi likely increased, resulting in an increase in network complexity.

### 4.3. Interaction between dissolved organic carbon and fungal network complexity governs the carbon mineralization

Comprehending the results of random forests and VPA, this study suggested that the interaction between DOC characteristics and microbial network, particularly fungal network complexity, was the dominant factors regulating C mineralization. The conclusion is consistent with the third hypothesis of the study. Dissolved organic carbon (DOC) is the most active component in SOC and provides a soluble organic substrate for heterotrophic microorganisms, and thus concentrations and components of DOC are essential factors affecting microbial community composition and function and vice versa (Zheng et al., 2021; Dou et al., 2022; Hu et al., 2022). The interaction between DOC components and soil microbial structure is the core of the C cycle. Microbial community composition (Schimel and Schaeffer, 2012) and specifically microbial network characteristics (Banerjee et al., 2016; Morrien et al., 2017) are increasingly considered to regulate soil C mineralization. In this study, increases in DOC concentrations and microbially derived compounds accounted for 18.3% of the variance in C mineralization. Meanwhile, the interaction between DOC characteristics and increased fungal network complexity accounted for over 60% of the variance in C mineralization (Figures 6C,D). Compare with less connected networks, highly connected networks may contribute to efficient C utilization (Morrien et al., 2017). The results of this study indicated that fungi were more active in C cycling with a potentially more connected network, especially when biochar-induced saprophytic fungi, such as *Cladorrhinum*, mineralize recalcitrant C (Ling et al., 2022). The results highlighted the importance of the interaction between DOC characteristics and fungal network complexity on C mineralization.

## 5. Conclusion

Our study showed that co-incorporation of green manure and biochar increased C mineralization, DOC concentrationsand microbially derived compounds, shifted microbial community composition, and increased fungal network complexity whereas decreased bacterial network complexity. The interaction between DOC characteristics and fungal network complexity was the dominant factor controlling C mineralization. The study highlighted that C1, i.e., the microbial humic acid-like component of DOC, influenced C mineralization by influencing fungal network complexity. In conclusion, the study showed how DOC-microbial network interaction-controlled C mineralization under the soil degradation improvement measure of co-incorporation of green manure and biochar. Additional studies should be conducted to evaluate whether the conclusion of the study applies to other soil types or under different climatic conditions.

## Data availability statement

The datasets presented in this study can be found in online repositories. The names of the repository/repositories and accession number(s) can be found at: Sequence Read Archive (SRA) in NCBI under Bioproject PRJNA933112.

## Author contributions

KC, XW, LF, WW, ML, and BS contributed intellectual input and assistance to this study and manuscript. BS and XW developed the research framework. KC and XW were responsible for investigation, formal analysis, and wrote the manuscript. LF, WW, and ML were responsible for software visualization, validation, and supervision. All authors contributed to the article and approved the submitted version.

## Conflict of interest

The authors declare that the research was conducted in the absence of any commercial or financial relationships that could be construed as a potential conflict of interest.

The reviewer XL declared a shared affiliation with the authors KC, XW, ML, and BS to the handling editor at the time of review.

## Publisher’s note

All claims expressed in this article are solely those of the authors and do not necessarily represent those of their affiliated organizations, or those of the publisher, the editors and the reviewers. Any product that may be evaluated in this article, or claim that may be made by its manufacturer, is not guaranteed or endorsed by the publisher.
