# Supplementary material for: Interaction between dissolved organic carbon and fungal network governs carbon mineralization in paddy soil under co-incorporation of green manure and biochar
Source: Front Microbiol. 2023 Aug 22;14:1233465. doi: 10.3389/fmicb.2023.1233465 (PMC10477716; doi:10.3389/fmicb.2023.1233465)
Supplement: Supplementary file 1 [file Table_1.DOCX]

**Supplementary Materials for**

**The interaction of DOC and fungal network governs the SOC mineralization in paddy soil under co-incorporation of green manure and biochar**

Kun Cheng ^1,2^, Xiaoyue Wang ^2, *^, Libo Fu ^3^, Wei Wang ^3^, Ming Liu ^2^, Bo Sun ^2, *^

***Corresponding authors:**

Xiaoyue Wang, Tel: +86 86881245, E-mail address: wangxy@issas.ac.cn (X. Wang);

Bo Sun, Tel: +86 86881282, E-mail address: bsun@issas.ac.cn (B. Sun).

**This PDF file includes:**

Table S1 to S3

Table S1

Basic characters of tested soil samples

|  | pH | SOC  (g·kg^-1^) | NH_4_^+^-N  (mg·kg^-1^) | NO_3_^-^-N  (mg·kg^-1^) | TN  (g·kg^-1^) | C/N | TP  (g·kg^-1^) | AP  (mg·kg^-1^) |
| --- | --- | --- | --- | --- | --- | --- | --- | --- |
| Soil | 7.65±0.06 | 20.96±1.03 | 10.32±0.95 | 0.42±0.12 | 1.97±0.16 | 10.58±0.60 | 0.56±0.25 | 89.37±5.02 |

Notes: Values was mean ± standard error. SOC, soil organic carbon; NH_4_^+^-N, ammonia nitrogen; NO_3_^-^-N, nitrate nitrogen; TN, soil total nitrogen; C/N,

soil organic carbon to total nitrogen ratio; TP, soil total phosphorus and AP, soil available phosphorus.

Table S2

Main nutrient contents of green manure and biochar

| Treatment | pH | DOC  (g·kg^-1^) | TC  (g·kg^-1^) | TN  (g·kg^-1^) | TP  (g·kg^-1^) | TK  (g·kg^-1^) | C/N |
| --- | --- | --- | --- | --- | --- | --- | --- |
| Biochar | 9.77±0.15 | 0.79±0.11 | 402.91±6.73 | 14.11±1.20 | 2.25±0.19 | 15.7±0.98 | 28.87±1.96 |
| Green manure | 5.73±0.28 | 135.50±7.60 | 495.99±21.55 | 32.92±2.47 | 2.89±0.32 | 33.35±1.97 | 15.13±0.52 |

Notes: Values was mean ± standard error. DOC, dissolved organic carbon; TC, total carbon; TN, total nitrogen; TP, soil total phosphorus;

TK, total potassium and C/N, total carbon to total nitrogen ratio.

Table S3

Rice biomass (dry weight) at harvest stage

| Treatment | Plant height  (cm) | Stem  (g·pot^-1^) | Leaf  (g·pot^-1^) | Grain  (g·pot^-1^) | Root  (g·pot^-1^) |
| --- | --- | --- | --- | --- | --- |
| NPK | 90.33±2.85a | 14.86±1.42b | 3.99±0.88b | 2.93±0.69b | 1.55±0.10a |
| GM | 94.31±4.07a | 21.90±3.48ab | 4.71±0.36ab | 5.46±0.38a | 1.59±0.19a |
| GMC | 97.30±2.38a | 28.39±2.61a | 6.68±0.36a | 6.22±0.72a | 1.91±0.22a |

Notes: Values within the same column followed by different letters indicate significant differences at *p* < 0.05.
